# Supplementary material for: MetaRibo-Seq measures translation in microbiomes
Source: Nat Commun. 2020 Jun 29;11:3268. doi: 10.1038/s41467-020-17081-z (PMC7324362; doi:10.1038/s41467-020-17081-z)
Supplement: Supplementary file 10 — Supplementary Data 7 [file 41467_2020_17081_MOESM10_ESM.zip › File2/Confidence_High_Taxonomy/328489_out.krona.html]

Javascript must be enabled to view this page.

members
magnitude
magnitudeUnassigned
count
unassigned
taxon
rank

328489\_out

6

superkingdom
2
6

phylum
1239
6

6
186801
class

order
186802
6

6
186803
family

6
39491
species

SRS077641\_contig\_number\_14190SRS1041133\_contig\_number\_27838SRS144714\_contig\_number\_29627SRS147977\_contig\_number\_10039SRS148196\_contig\_number\_contig-100\_49.247248SRS893252\_contig\_number\_16946
